# Meta analysis: HPV and p16 pattern determines survival in patients with HNSCC and identifies potential new biologic subtype

**DOI:** 10.1038/s41598-017-16918-w

**Published:** 2017-12-01

**Authors:** Andreas E. Albers, Xu Qian, Andreas M. Kaufmann, Annekatrin Coordes

**Affiliations:** 1 Department of Otorhinolaryngology, Head and Neck Surgery, Berlin Institute of Health, Charite – Universitätsmedizin Berlin, corporate member of Freie Universität Berlin, Humboldt-Universität zu Berlin, Campus Benjamin Franklin, Berlin, Germany; 2 Clinic for Gynecology, Charité – Berlin Institute of Health, Charite – Universitätsmedizin Berlin, corporate member of Freie Universität Berlin, Humboldt-Universität zu Berlin, Campus Benjamin Franklin, Berlin, Germany

## Abstract

Consistent discrepancies in the p16/HPV-positivity have been observed in head and neck squamous cell carcinoma (HNSCC). It is therefore questionable, if all HPV^+^ and/or p16^+^ tested cancers are HPV-driven. Patients down-staged according to the HPV-dependant TNM are at risk for undertreatment and data in clinical trials may be skewed due to false patient inclusion. We performed a meta-analysis to classify clinical outcomes of the distinct subgroups with combined p16 and HPV detection. 25 out of 1677 publications fulfilled the inclusion criteria. The proportion of the subgroups was 35.6% for HPV^+^/p16^+^, 50.4% for HPV^−^/p16^−^, 6.7% for HPV^−^/p16^+^ and 7.3% for HPV^+^/P16^−^. The HPV^+^/p16^+^ subgroup had a significantly improved 5-year overall-survival (OS) and disease-free-survival in comparison to others both for HNSCC and oropharyngeal cancers. The 5-year OS of the HPV^−^/p16^+^ HNSCC was intermediate while HPV^+^/p16^−^ and HPV^−^/p16^−^ had the shortest survival outcomes. The clearly distinct survival of HPV^−^/p16^+^ cancers may characterize a new relevant HPV-independent subtype yet to be biologically characterized. The possibility also exists that in some HPV^+^/p16^+^ cancers HPV is an innocent bystander and p16 is independently positive. Therefore, in perspective, HPV-testing should distinguish between bystander HPV and truly HPV-driven cancers to avoid potential undertreatment in HPV^+^ but non-HPV-driven HNSCC.

## Introduction

Head and neck squamous cell carcinoma (HNSCC) is the sixth most common cancer worldwide^1^. From an epidemiological perspective, exogenous carcinogen-exposure (tobacco and alcohol consumption)-induced HNSCC is declining, while human papillomavirus (HPV) infection-driven HNSCC is increasing in younger individuals in recent years^2^. Due to a lack of screening-test the incidence of HPV^+^ HNSCC is increasing, while cervical cancer is decreasing in the USA^3^ and Europe for example in Germany^4^ and France^5^. If HNSCC is HPV^+^ this may be, because HPV drives carcinogenesis and can then be considered as causal, or HPV is detected as an innocent bystander of concurrent infection^6^. This bystander infection can be imagined as an infection located next to the tumor and co-sampled with the tumor material or as an integrated virus copy that has been silenced e.g. by promoter methylation or gene deletion with no active gene transcription. Thus, only if the expression of HPV-related oncogenes is detected, the tumor is proven to be HPV-driven^6–8^ indicated by the existence of HPV^+^/p16^−^ cases. Thus, unless HPV-oncogene expression has been determined the tumor should be referred to as HPV-associated.

HPV^+^ tumors have been identified in many regions of head and neck^2,9^, especially in oropharyngeal squamous cell carcinomas (OPSCC)^10,11^. These tumors differ from HPV-unrelated cancers at the molecular level^12–15^ and likely, as a consequence, have a much more favorable prognosis, despite the generally more advanced stage at presentation^16^. Given by the nature of HPV-driven tumors, the recently released 8th Edition of the American Joint Committee on Cancer (AJCC) created a separate staging algorithm for high-risk-HPV-associated cancer of the oropharynx distinguishing it from HPV^−^ OPSCC^17^. HPV^+^ OPSCC, that used to be advanced stage are now categorized into lower stage. This gives a much more accurate and reasonable prediction of survival for patients with HPV^+^ OPSCC.

Preclinical studies also demonstrated that biological features depending on tumor HPV status would influence the effectiveness of treatment. HPV-driven tumors respond better to chemotherapy and X-ray or proton therapy than HPV^−^ tumors^18–20^. Because of the differences in biological features and prognosis, individually optimized therapy for patients with HPV-driven tumors would minimize treatment-related toxicity and improve outcomes. Consequently, de-escalated treatment protocols are under investigation.

Most importantly, for a correct classification a reliable distinction between the different entities is crucial. HPV-DNA testing and p16^ink4a^ (cyclin-dependent kinase 2a) immunohistochemical staining are both well-established methods in identifying HPV^+^ tumors and often regarded delivering equal information on HPV positivity. This is due to the correlation of high-risk HPV E7 expression and in consequence an upregulation of p16. Since, p16 immunohistochemical staining is inexpensive, convenient in use, and the interpretation of results is established it is widely used for detection of HPV-related HNSCC. Therefore, in the modified 8th AJCC/UICC, p16 was recommended as HPV surrogate marker with the cutoff point for diffuse (≥75%) overexpression in a histological section and at least moderate (+2/3) staining intensity^17^. Although both p16 overexpression and HPV-DNA-positivity have shown their independent prognostic value, as we summarized before^21^, there are p16^+^/HPV^−^ and p16^−^/HPV^+^ subgroups in which surprisingly different prognosis relative to p16- and HPV-status was observed. Survival of patients with HNSCC was better if associated with HPV^+^/p16^+^ or HPV^−^/p16^+^. Therefore, in addition to the HPV-related prognostic feature, the biological relevance of p16 independent of HPV infection is currently of interest and under investigation, possibly describing another subgroup of HNSCC with a role of p16 in HPV-independent HNSCC.

In this meta-analysis, we included all current clinical studies and evaluated the clinical relevance of HPVDNA-positivity and p16 overexpression in HNSCC. Current observations in elucidating the biological role of p16 in HPV^+^ and HPV^−^ tumors were also discussed.

## Methods

### Selection criteria and literature search strategy

Four database searches were performed for publications that statistically analysed subgroup survival after detection of both HPV and p16 markers in PubMed (http://www.ncbi.nlm.nih.gov/pubmed), OVID (www.ovid.com), EMBASE (www.embase.com), and Wanfang (www.wanfangdata.com.cn). This search included publication dates up to April 20, 2017, adding an additional two years to the previously performed literature search^21^. We searched for the terms “HPV, p16, head neck”. We also included references quoted in original or review articles that may not have been found during the initial literature search. We screened the articles and included all studies of HNSCC patients which investigated survival rates by the p16 and HPV status of the tumor. Our search strategy was performed in accordance with PRISMA criteria and registered in the PROSPERO register (CRD42017062330). We excluded studies that met the following criteria: missing patient survival information, evaluation of only one marker (HPV or p16), non-HNSCC primary cancer (e.g., nasopharyngeal carcinoma, skin cancer, pre-cancer), cell culture or animal models, and reviews or case reports. We also excluded studies with duplicate patient data from the same or similar populations (based on the authors’ names and institutions) in a second round selection process. Where this occurred, we selected the study which was either more recent or had larger patient numbers. We also excluded studies with insufficient survival data. Finally, we included studies with the following criteria: (1) the numerical portion of the subgroups HPV^+^/p16^+^ versus HPV^−^/p16^−^ versus HPV^+^/p16^−^ versus HPV^−^/p16^+^ in HNSCC patients; (2) the numerical survival data of these subgroups (Hazard ratio (HR); overall survival (OS); disease free survival (DFS)) or Kaplan-Meier curves of the subgroups of OS or DFS.

### Data extraction

Two authors (A.C. and A.E.A.) extracted the relevant data from the selected publications according to the aforementioned inclusion criteria^21^. In the case of any discrepancies, we re-analysed the study and the two authors reached a consensus decision. We extracted all relevant information from the studies, including: author, publication date, study timeframe, country, tumor stage and localisation, number of patients, study design, data on alcohol and tobacco consumption, number of HPV positive and negative patients, number of patients included in the subgroups HPV^+^/p16^+^, HPV^−^/p16^-^, HPV^+^/p16^−^, or HPV^−^/p16^+^, HPV subtypes, HR-status, 5-year OS or DFS of the subgroups, p16 and HPV detection method. We used GraphClick (Version 3.0.2, Arizona Software 2010, www.arizona-software.ch/graphclick) for data processing in studies where the OS or DFS was displayed as Kaplan-Meier plot.

### Statistical analysis

The OS and DFS of all subgroups was evaluated using relative risk (RR)^21^, calculating summary RR estimates and 95% confidence intervals (CI) using maximum-likelihood methods for linear mixed models. We assessed study heterogeneity using a chi-squared based Q test. An absence of heterogeneity between the studies was indicated by a p-value greater than 0.05. Existing heterogeneity was examined using the I2 index in the meta-analysis, which was represented as a percentage value between 0 and 100. We initially applied a fixed-effects model (Mantel-Haenszel method and chi-squared test) to the data. Where there was significant heterogeneity, we used the random-effects model (DerSimonian-Liard method). We examined the RR of the 5-year OS and DFS of all subgroups correlating with the HPV^+^/p16^+^ and HPV^−^/p16^−^ groups, depending on the data we extracted from each publication. In cases where the HR was described in the studies, we performed the same analysis. We compared all of the studies using a forest plot.

Publication bias was examined using a funnel plot. We used the R Version 3.1.0 (R Core Team 2014) computing environment for all statistical analyses^22^.

### Data Availability

The datasets generated during and/or analysed during the current study are available from the corresponding author on reasonable request.

## Results

### Study characteristics

**Figure 1:**
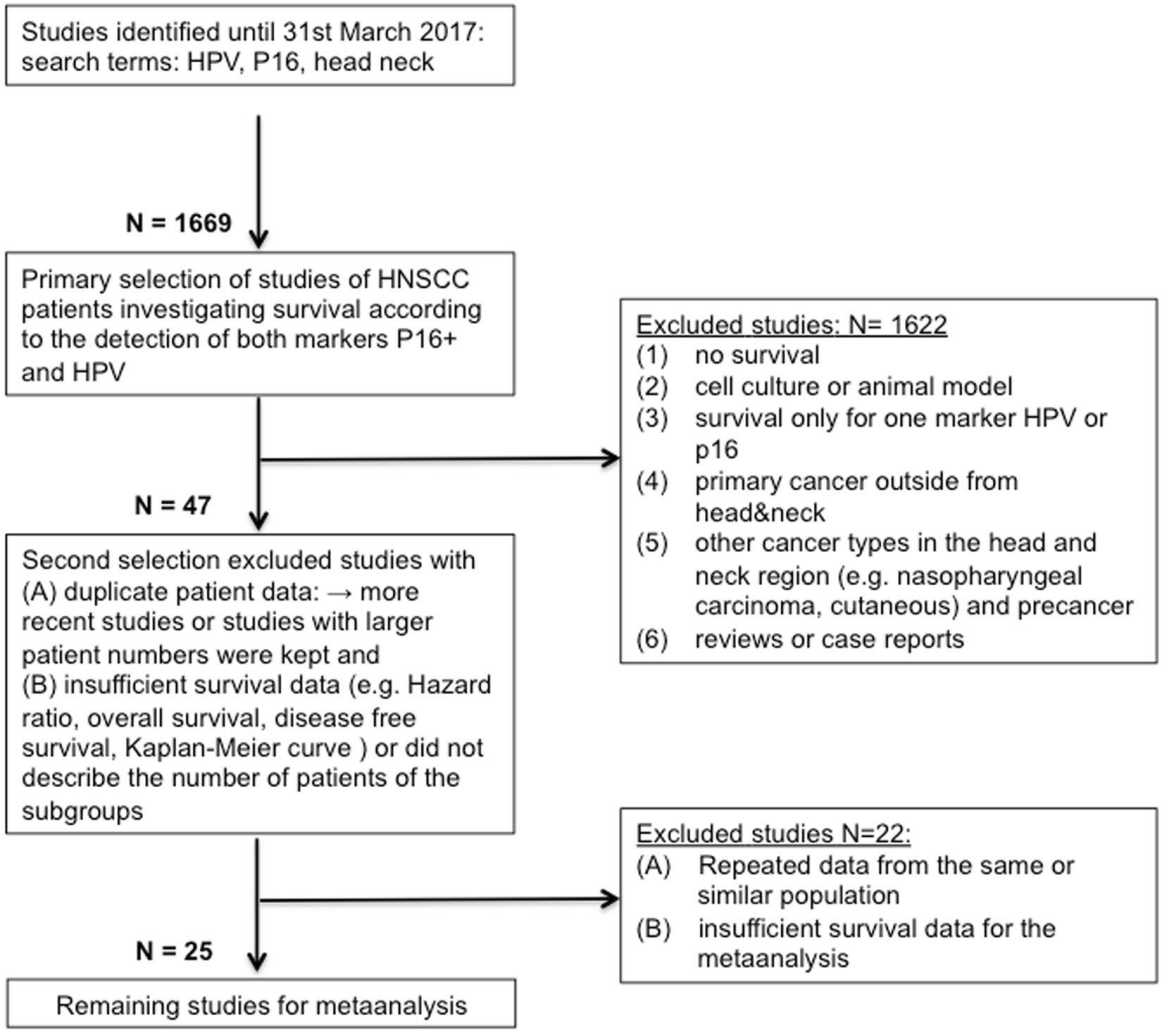
Literature search strategy and selection of articles.

The initial search yielded 1677 citations (Fig. 1). 25 articles met the inclusion criteria^23–47^ (Fig. 1 and Table 1) including a total of 6852 patients, with patients per study ranging between 34 and 1542. The previous study had covered 18 studies and enrolled 4424 patients^21^. An updated literature search located 7 further studies including 2428 patients. However, the HPV/p16-status for some of these patients is missing, therefore we finally analyzed the data of 5131 study patients (range, 34–1479 patients per study). The main characteristics of the eligible studies were summarized in Tables 1–3. We have investigated 18 articles with studies that enrolled patients with UICC tumor stage I-IV, and 13 studies separately with OPSCC (Table 1). 20 studies performed HPV detection after preceding polymerase chain reaction (PCR) and 5 performed *in situ* hybridization (ISH) without earlier PCR. The identified subgroups discriminated by HPV and p16-status are represented in Table 2. The proportions of each subgroup were estimated from 22 studies, which clearly have patient numbers^23–28,30–35,37–44,46,47^. In all studies combined, the subgroup of HPV^+^/p16^+^ was 35.6%, of HPV^−^/p16^−^ 50.4%, of HPV^−^/p16^+^ 6.7% and of HPV^+^/p16^−^ 7.3%. In the studies that only investigated OPSCC, the subgroup of HPV^+^/p16^+^ was 44.9%, of HPV^−^/p16^−^ 44.4%, of HPV^−^/p16^+^ 5.4% and of HPV^+^/p16^−^ 5.3%. The studies investigating correlations between HPV-status and clinicopathological characteristics like tumor size, lymph node involvement, smoking etc. summarized in Table 3. This analysis includes 10 European, 9 North American (USA and Canada) and 6 Asian studies.

**Table 1:** Main characteristics of the eligible studies.

	Study [Year]	Time interval of collected data	Total	Men/ women	UICC stage	Localization	HPV subtypes	Mean age [Year]
1	Wittekindt C^23^	—	34	25/9	I-IV	OPSCC	16, 33	61
2	Smith EM^24^	1994–2004	301	188/113	I-IV	all	16, 33, 18	
3	Kong CS^25^	—	99	69/13	II-IV	all	16, 18, 33	
4	Weinberger PM^26^	1980–1999 (Yale), 2004–2007 (Georgia)	140	106/34	I-IV	all	16, 18	60.3
5	Heath S^27^	2004–2007	83	48/35	I-IV	all	HPV16	64
6	Park WS^28^	2002–2007	93	80/13	I-IV	OPSCC	16, 18, 33	62.1
7	Holzinger D^29^	1990–2008	199	146/50	I-IV	OPSCC	16, 18, 33, 35	57
8	Liang C^30^	1999–2003	844		I-IV	all	16	
9	Park K^31^	2000–2008	142	73/6	III-IV	OPSCC		54
10	Evans M^32^	2001–2006	147	104/34	I-IV	all	16, 18, 33, 56	58.1
11	Stephen JK^34^	1986–2003	80	66/14	I-IV	all	16	
12	Rietbergen MM^35^	2000–2006	841		I-IV	OPSCC	16, 35, 33, 18, 45, 58	
13	Huang H, 2013^36^	1999–2009	66		I-IV	OPSCC	16,33,11,35,52,54,58	59
14	Semrau R^41^	2000–2008	52	42/10	III-IV	OPSCC	16, 18, 33, 35	56.3
15	Melkane AE^33^	2007–2009	133	94/39	I-IV	OPSCC	16, 18, 33	59
16	Salazar CR^37^	—	158	110/43	I-IV	OPSCC	16	
17	Heiduschka G^39^	2002–2013	103	46/17	II-IV	OPSCC		60.5
18	Chung CH^40^	—	683		III-IV	all non-OPSCC		58
19	Ramshankar V^42^	1995–2007	167	108/48	I-II	HNSCC	16	
20	Xu Y^38^	2004–2013	278	229/27	I-IV	all	11, 16, 33	
21	Liu JC^43^	1990–2010	44	30/14	I-IV	OPSCC	16, 35	64
22	Saito Y^44^	2004–2012	167	130/20	I-IV	all		64
23	Descamps G^45^		218	173/45	II-IV	HNSCC		
24	Mazul AL^46^	2002–2006	238		I-IV	OPSCC	6,11,16,18, 26,31,33,35,39,58,59,82	
25	Gronhoj Larsen C^47^	2000–2014	1542		I-IV	OPSCC		

Footnotes: ICC, Yale, Georgia.

Abbreviations: Union for International Cancer Control, UICC; oropharyngeal squamous cell carcinoma, OPSCC; human papillomavirus, HPV.

**Table 2:** Number of patients according to the subgroups depending on the detection of HPV and p16.

	Study [Year]	Patients extracted for analysis	HPV^+^/p16^+^	HPV^−^/ P16^−^	HPV^−^/ p16^+^	HPV^+^/ p16^−^
1	Wittekindt C^23^	34	16	16	0	2
2	Smith EM^24^	301	62	175	43	19
3	Kong CS^25^	82	30	36	10	6
4	Weinberger PM^26^	102	25	44	0	33
5	Heath S^27^	60	18	34	8	0
6	Park WS^28^	93	46	40	0	7
7	Holzinger D^29^	178	42	73	12	50
8	Liang C^30^	121	31	43	7	40
9	Park K^31^	79	50	12	13	4
10	Evans M^32^	138	69	59	4	6
11	Stephen JK^34^	80	12	31	9	28
12	Rietbergen MM^35^	723	152	545	26	0
13	Huang H^36^	66	9	43	12	2
14	Semrau R^41^	52	13	33	4	2
15	Melkane AE^33^	126	61	40	4	21
16	Salazar CR^37^	36	15	17	3	1
17	Heiduschka G^39^	63	25	25	13	0
18	Chung CH^40^	273	20	213	33	7
19	Ramshankar V^42^	156	10	73	14	59
20	Xu Y^38^	256	9	239	8	0
21	Liu JC^43^	44	13	11	0	20
22	Saito Y^44^	150	48	89	10	3
23	Descamps G^45^	213	17	170*	**	26
24	Mazul AL^46^	226	140	59	7	20
25	Gronhoj Larsen C^47^	1479	810	503	91	75
	In total	5131	1726	2623	331	431

*Number of patients representing two different subgroups. **Not determined.

**Table 3:** Selected characteristics of the 25 studies.

Study design	Case-control	16/25 (64.0%)
	Cohort	9/25 (36.0%)
Region	US	9/25 (36.0%)
	Europe	10/25 (40.0%)
	Asia	6/25 (24.0%)
HPV detection method	ISH (without PCR)	5/25 (20.0%)
	PCR-based	20/25 (80.0%)
	L1-target	15/20 (75%)
	E6/E7-target	5/20 (25%)
Rate of HPV^+^ HNSCC	Tumor size (T)	5/9 (55.6%)
	Cervical lymph node metastases (N)	6/9 (66.7%)
	Advanced UICC tumor stage	6/11 (54.5%)
	OPSCC localisation	7/7 (100%)
	Low grading of cancer	5/7 (71.4%)
	Non-drinking	4/11 (36.4%)
	Non-smoking	8/14 (57.1%)
	p16 ^+^	13/16 (81.3%)
	Younger age	7/12 (58.3%)
	Gender	1/9 (11.1%)

Abbreviations: Union for International Cancer Control, UICC; oropharyngeal squamous cell carcinoma, OPSCC; squamous cell carcinoma, SCC.

### 5-year OS and HPV/p16 subgroup status

**Figure 2:**
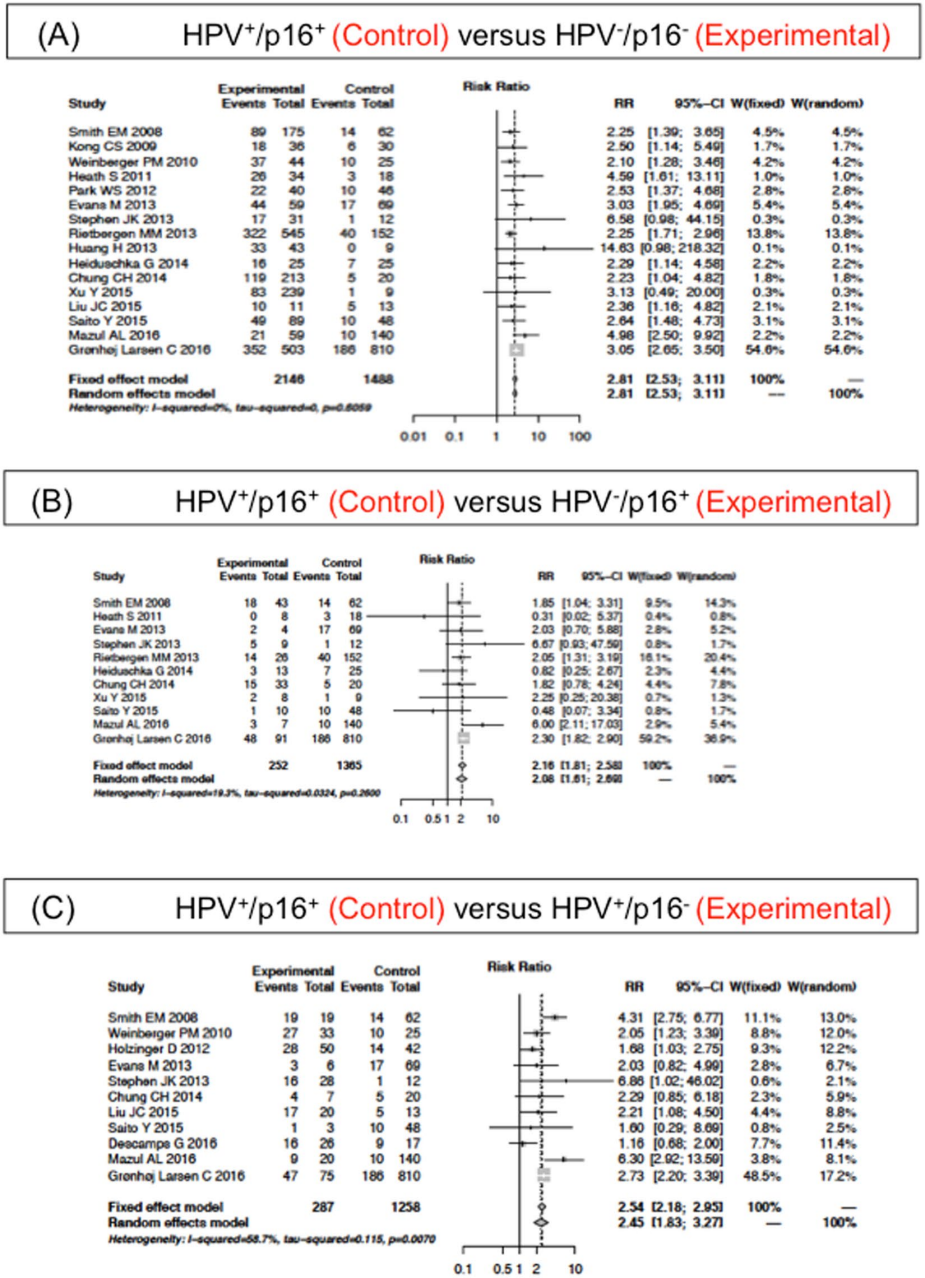
Adjusted Relative Risk (RR) of the 5-year overall survival (OS) compared to the HPV^+^/p16^+^ subgroup. Forest plot of RR among included studies for the 5-year OS of the HPV^+^/p16^+^ subgroup compared to (**A**) HPV^−^/p16^−^, (**B**) HPV^−^/p16^+^ and (**C**) HPV^+^/p16^−^. Combined RR was calculated by a random mode.

Pooled outcome (5-year OS) results of 19 studies for all four distinct HPV/p16 subgroups can be seen in Fig. 2. For the 5-year OS investigation, 16 of 19 studies (3634 patients) contained data suitable for subgroup HPV^+^/p16^+^ and HPV^−^/p16^−^ analysis. Figure 2A depicts a forest plot of this meta-analysis and demonstrates that subgroup HPV^+^/p16^+^ is associated with improved OS (fixed effects model; RR of 2.81; 95% CI 2.53–3.11; P = 0.61). Eleven studies that enrolled 1617 patients have data for subgroup HPV^+^/p16^+^ and HPV^−^/p16^+^ analysis, and 11 studies that enrolled 1545 patients for subgroup HPV^+^/p16^+^ and HPV^+^/p16^−^. As shown in Fig. 2B and C, a forest plot demonstrates that HPV^+^/p16^+^ has an improved OS compared with both the HPV^−^/p16^+^ (fixed effects model; RR of 2.16; 95%CI 1.81–2.58; P = 0.26) and the HPV^+^/p16^−^ (random effects model; RR of 2.45; 95%CI 1.83–3.27; P = 0.007).

**Figure 3:**
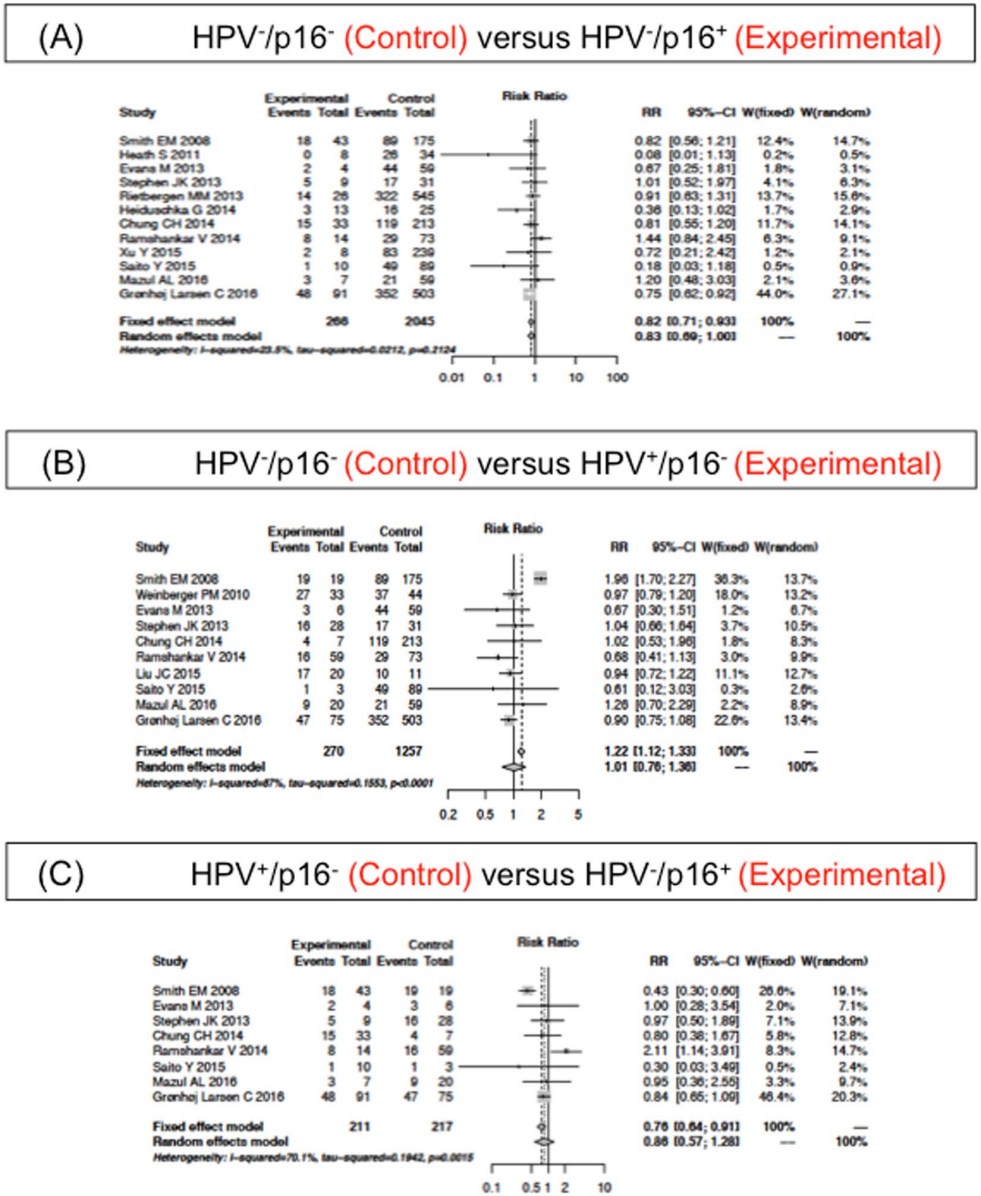
Adjusted Relative Risk (RR) of the 5-year overall survival (OS) compared to the HPV^−^/p16^−^ subgroup. Forest plot of RR among included studies for the 5-year OS of the HPV^−^/p16^−^ subgroup compared to (**A**) HPV^−^/p16^+^ and (**B**) HPV^+^/p16^−^. (**C**) Forest plot of RR compares HPV^−^/p16^+^ and HPV^+^/p16^−^. Combined RR was calculated by a random mode.

Twelve of 19 studies (2311 patients) displayed data for the 5-year OS of subgroup HPV^−^/p16^−^ and HPV^−^/p16^+^ and 10 studies (1527 patients) of subgroup HPV^−^/p16^−^ and HPV^+^/p16^−^. The forest plot in Fig. 3B shows the 5-year OS of HPV^−^/p16^−^ does not significantly differ from the HPV^+^/p16^−^ (random effects model; RR of 1.01; 95%CI 0.76–1.36; P < 0.0001). However, the 5-year OS of the HPV^−^/p16^−^ subgroup was inferior compared to the HPV^−^/p16^+^ (fixed effects model; RR of 0.82; 95%CI 0.71–0.93; P = 0.21; Fig. 3A).

Additionally, 8 studies (428 patients) reported the 5-year OS of subgroup HPV^+^/p16^−^ and HPV^−^/p16^+^ (Fig. 3C). There was no significant difference between the two subgroups. However, when one study (Ramshankar *et al*.^42^) was excluded, the 5-year OS of the subgroup HPV^+^/p16^−^ was inferior compared with the HPV^−^/p16^+^ (fixed effects model; RR of 0.7, 95%CI: 0.58–0.84, P = 0.06). The Ramshankar *et al*.^42^ study reported a small correlation between HPV16-DNA and p16 expression in oral tongue SCC patients. Those patients with p16 overexpression showed an increased risk of death and disease recurrence regardless of their HPV16-DNA status.

### 5-year OS of the HPV/p16 subgroups for cancers of oropharyngeal origin

**Figure 4:**
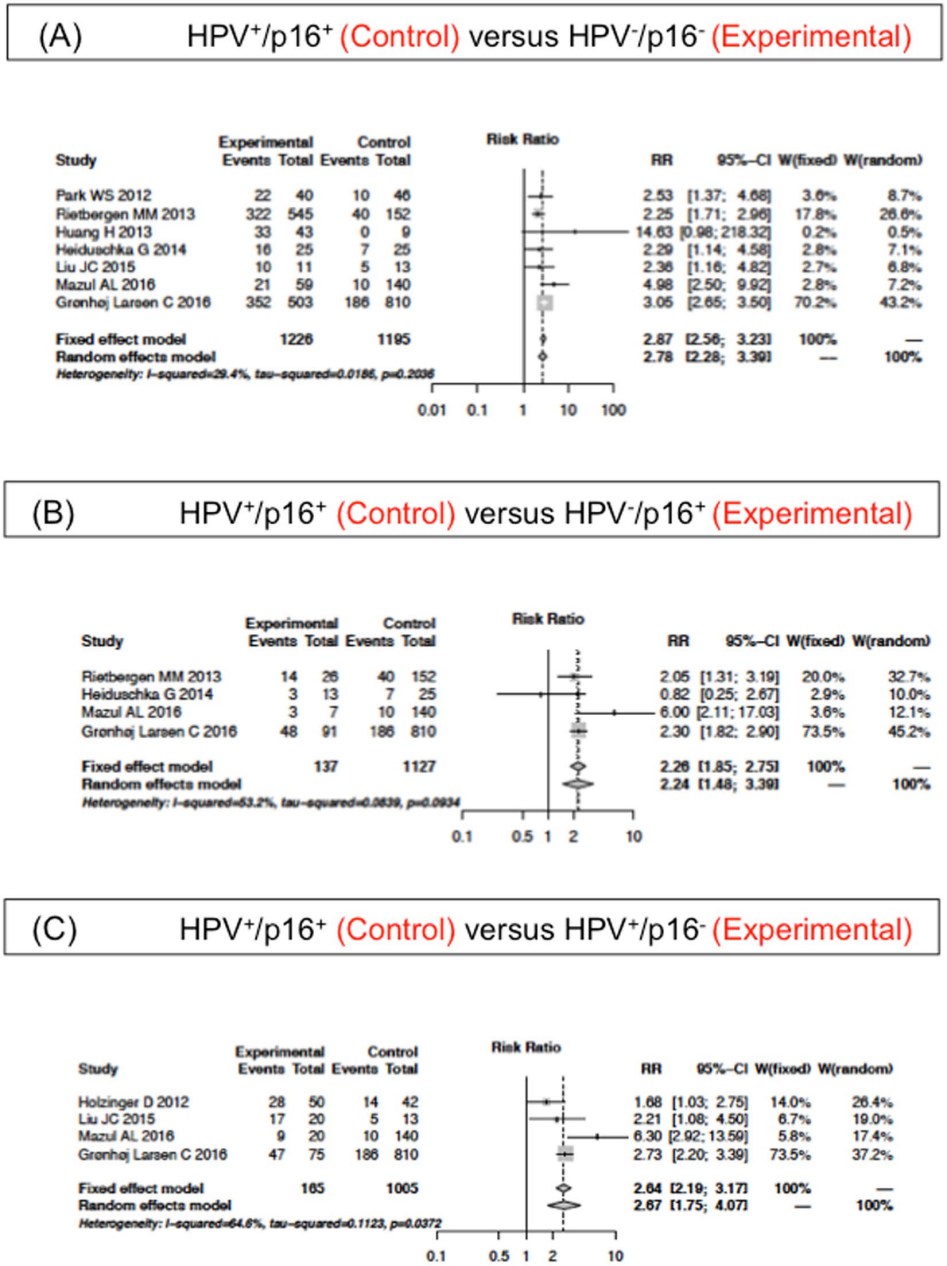
Adjusted Relative Risk (RR) of the 5-year overall survival (OS) compared to the HPV^+^/p16^+^ subgroup in patients with oropharyngeal cancer origin. Forest plot of RR among included studies for the 5-year OS of the HPV^+^/p16^+^ subgroup compared to (**A**) HPV^−^/p16^−^, (**B**) HPV^-^/p16^+^ and (**C**) HPV^+^/p16^−^. Combined RR was calculated by a random mode.

**Figure 5:**
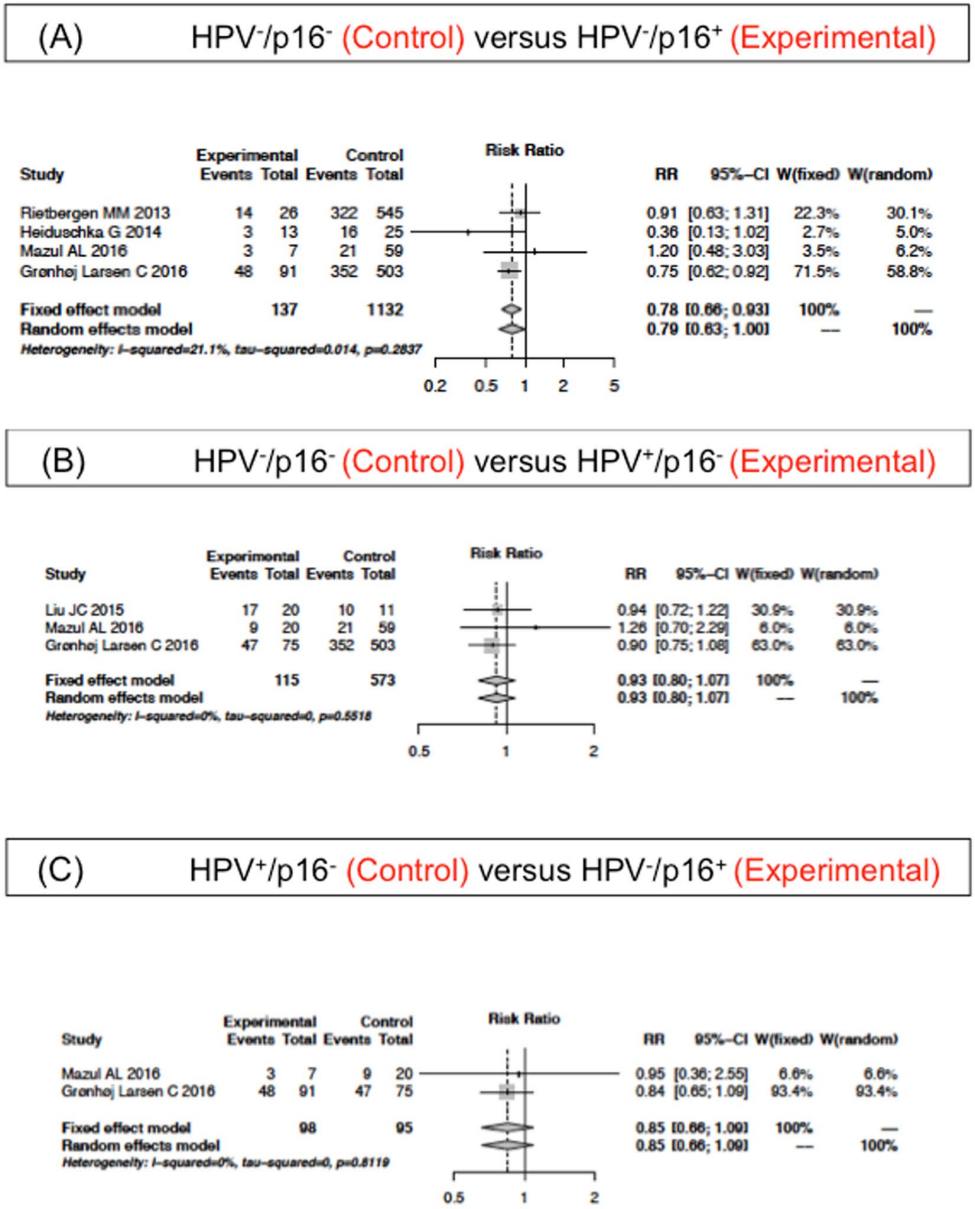
Adjusted Relative Risk (RR) of the 5-year overall survival (OS) compared to the HPV^−^/p16^−^ subgroup in patients with oropharyngeal cancer origin. Forest plot of RR among included studies for the 5-year OS of the HPV^−^/p16^−^ subgroup compared to (**A**) HPV^−^/p16^+^ and (**B**) HPV^+^/p16^−^. Combined RR was calculated by a random mode.

5-year OS in all distinct HPV/p16 subgroups in cancer of oropharyngeal origin was determined in 9 studies. The subgroup HPV^+^/p16^+^ showed a better survival than the HPV^−^/p16^−^ (fixed effects model; RR of 2.87; 95% CI 2.56–3.23; P = 0.20), the HPV^−^/p16^+^ (fixed effects model; RR of 2.26; 95% CI 1.85–2.75; P = 0.09) and the HPV^+^/p16^−^ (random effects model; RR of 2.67; 95% CI 1.75–4.07; P = 0.04). Figure 4A–C show the forest plots of the meta-analysis for each comparison. Four studies included data on the 5-year OS of the subgroup HPV^−^/p16^−^ and the HPV^−^/p16^+^, and 3 studies of the HPV^−^/p16^−^ and the HPV^+^/p16^−^ subgroup. The forest plot in Fig. 5A demonstrates the superior 5-year OS of HPV^−^/p16^+^ compared with HPV^−^/p16^−^ (fixed effects model; RR of 0.78; 95%CI 0.66–0.93; P = 0.28). The subgroup HPV^−^/p16^−^ did not differ significantly to the HPV^+^/p16^−^ (fixed effects model; RR of 0.93; 95%CI 0.80–1.07; P = 0.55; Fig. 5B). It was not possible to meta-analyze the subgroup HPV^+^/p16^−^ and the HPV^−^/p16^+^ as there were only two studies presenting these data. The analysis of OPSCC patients revealed a similar 5-year OS of the subgroups in studies investigating HNSCC.

### HR for OS of the HPV/p16 subgroups

We could determine the OS-HR from 12 studies. Five studies used HPV^+^/p16^+^ and 7 studies used HPV^−^/p16^−^ as reference markers. We summarize the results of the individual meta-sub-analyses in Table 4. The HR for the OS of the subgroup HPV^+^/p16^+^ was significantly increased compared to the HPV^−^/p16^−^, being irrespective of whether HPV^+^/p16^+^ or HPV^−^/p16^−^ was used as reference values. The HR for the better OS of the subgroup HPV^+^/p16^+^ was significantly increased compared to the HPV^−^/p16^+^ and the HPV^+^/p16^−^, respectively. The meta-analyses of the HRs for the OS of the subgroup HPV^−^/p16^−^, the HPV^−^/p16^+^ and the HPV^+^/p16^−^ included only 2 and 4 studies, respectively. Because the HRs of these sub-analyses didn’t show significant differences, we are unable to draw any general conclusion due to the limited data.

**Table 4:** Meta-analyses on the hazard ratio of the overall survival of the subgroups of HPV^+/−^ and p16^+/−^.

	Number of studies	Total patient number	Number of patients of the control versus the experimental group	Fixed effect model HR (95%-CI), p value	Random effects model HR (95%-CI), p value	Quantifying heterogeneity	References
HPV^−^/p16^−^ versus HPV^+^/p16^+^	6	425	206/219	0.15 (0.15–0.15); p < 0.0001	0.19 (0.05–0.69); p = 0.0122	tau^2^ = 2.68; H = 949.3 (945.0–953.6) I^2^ = 100%; p < 0.0001	25,30,32,33,37,43
HPV^+^/p16^+^ versus HPV^-^/p16^-^	5	1768	971/797	4.60 (4.51–4.70); p < 0.0001	3.49 (1.93–6.31); p < 0.0001	tau^2^ = 0; H = 9.1 (7.6–11.0) I^2^ = 98.8%; p = 0.6005	24,26,28,34,47
HPV^+^/p16^+^ versus HPV^−^/p16^+^	3	1045	900/145	2.70 (2.59–2.82); p < 0.0001	3.3007 (1.4004; 7.7797); p < 0.0001	tau^2^ < 0.0001; H = 1.0 (1.0–1.0); I^2^ = 0%; p = 0.90	24,34,47
HPV^−^/p16^−^ versus HPV^−^/p16^+^	2	75	55/20	1.20 (0.73–1.99); p = 0.48	1.20 (0.73–1.99); p = 0.48	tau^2^ = 0; H = 1.0; I^2^ = 0%; p = 0.89	30,31
HPV^+^/p16^+^ versus HPV^+^/p16^-^	4	1080	925/80	4.09 (3.59–4.67) p < 0.0001	4.09 (3.59–4.67); p < 0.0001	tau^2^ = 0; H = 1.0 (1.0–1.0); I^2^ = 0%; p = 0.97	24,26,34,47
HPV^−^/p16^−^ versus HPV^−^/p16^−^	4	191	106/85	0.619 (0.60–0.64); p < 0.0001	0.76 (0.46–1.26); p = 0.29	tau^2^ = 0.20; H = 11.3 (9.4–13.6); I^2^ = 99.2%; p < 0.0001	30,31,33,43

Abbreviations: hazard ratio, HR.

### 5-year DFS in the distinct HPV/p16 subgroups

Eleven studies investigated 5-year DFS. We summarize the individual meta-analyses of the different subgroups in relation to HPV and p16 status individually in Table 5. Eight studies enrolled 2807 patients and had suitable data for meta-analyzing the 5-year DFS of the subgroups HPV^+^/p16^+^ and HPV^−^/p16^−^. The subgroup HPV^+^/p16^+^ showed a significantly improved 5-year DFS (fixed effects model; RR 1.96; 95% CI 1.73–2.22; P = 0.21). The 5-year DFS of the subgroup HPV^+^/p16^+^ was also significantly improved compared to the HPV^−^/p16^+^ and the HPV^+^/p16^−^. The 5-year DFS of the subgroup HPV^−^/p16^−^ did not differ significantly from the HPV^−^/p16^+^ and the HPV^+^/p16^−^, even when the Ramshankar *et al*.^42^ study was excluded (fixed effects model; RR 0.87; 95% CI 0.72–1.05; P = 0.095 and random effects model; RR 1.44; 95% CI 0.87–2.38; p < 0.001). Additionally, 4 studies enrolling 342 patients had the data of HPV^+^/p16^−^ and HPV^−^/p16^+^ patients for a 5-year DFS meta-analysis. Both subgroups did not differ to each other significantly, even when the Ramshankar *et al*.^42^ study was excluded. The DFS-HR was investigated in 6 studies^24–26,28,32,33^. The subgroup HPV^+^/p16^+^ was associated with a significantly improved 5-year DFS (random effects model; RR 2.63; 95% CI 2.60–2.67; P < 0.001).

**Table 5:** 5-year disease free survival of the subgroups of HPV^+/−^ and p16^+/−^.

	Number of studies	Total patient number	Number of patients of the control versus the experimental group	Fixed effect model HR (95%-CI); p-value	Random effects model HR (95%-CI)	Quantifying heterogeneity	References
HPV^+^/p16^+^ versus HPV^−^/p16^-^	8	2807	1196/1611	1.96 (1.73–2.22); p = 0.21	2.00 (1.69–2.36)	tau^2^ = 0.0146; I^2^ = 27.1%	24–26,32,35,40,41,47
HPV^+^/p16^+^ versus HPV^-^/p16^+^	4	1253	1059/194	1.64 (1.31–2.06); p = 0.21	1.57 (1.16–2.11)	tau^2^ = 0.0316; I^2^ = 34.2%	24,35,40,47
HPV^+^/p16^+^ versus HPV^+^/p16^−^	6	258	991/210	1.67 (1.39–2.02); p < 0.001	1.99 (1.20–3.29)	tau^2^ = 0.3212; I^2^ = 84.8%	24,26,29,40,45,47
HPV^-^/p16^−^ versus HPV^−^/p16^+^	5	1720	1512/208	0.97 (0.89–1.06); p = 0.09	0.90 (0.74–1.09)	tau^2^ = 0.0216; I^2^ = 50.5%	24,35,40,42,47
HPV^-^/p16^−^ versus HPV^+^/p16^−^	5	1204	1011/193	1.03 (1.00–1.06); p < 0.001	1.33 (0.84–2.11)	tau^2^ = 0.2494; I^2^ = 96%	24,26,40,42,47
HPV^+^/p16^−^ versus HPV^-^/p16^+^	4	342	160/182	0.96 (0.87–1.05); p < 0.001	0.64 (0.35–1.19)	tau^2^ = 0.3328; I^2^ = 89,5%	24,40,42,47

Abbreviations: disease free survival, DFS.

### Sensitivity analysis

Due to the low numbers of eligible articles, we performed a sensitivity analysis to test a possible bias on the 5-year OS of the subgroups HPV^+^/p16^+^ and HPV^-^/p16^-^ based on where the study was performed by continent. The results from sub-meta-analyses in the region of the North America^24–26,34,40,43,46^, Europe^27,32,35,39,47^, and Asia^28,36,38,44^ were comparable with the complete meta-analyses including all regions (p > 0.05) (Fig. 2a). The fixed effect model was used in all sub meta-analyses (data not shown) due to non-significant heterogeneity, indicating statistically robust results. The RR and CI were essentially unchanged in comparison with the whole meta-analyses.

We also performed a sensitivity analysis assessing the possible bias resulting from the HPV detection methods. We divided the meta-analysis of the 5-year OS of the subgroup HPV^+^/p16^+^ and HPV^−^/p16^−^ into two groups classified by HPV detection methods: one group using PCR^25–28,32,34,35,38,43,46,47^ and one group using ISH without PCR^36,39,40,44^. These two meta-analyses showed comparable results to the complete data meta-analyses (Fig. 2a). Again, the fixed effect model was used (data not shown) due to non-significant heterogeneity (p > 0.05). The RR and CI were essentially unchanged compared with the whole data meta-analyses.

In order to test for a bias introduced as systematic error (due to low sensitivity of the HPV detection (false HPV^−^) inherent to some detection methods (e.g. ISH) or testing of only individual HPV types (only HPV16 or 18; missed other types)), we investigated the HPV^−^/p16^+^ subgroup further, by excluding the following studies^26,27,31,33,34,36,37,39,40^. However, meta-analysis of the 5-year OS of the subgroup HPV^+^/p16^+^ and HPV^-^/p16^+^ (fixed effects model; RR of 2.23; 95% CI 1.85–2.68; P = 0.37)^24,32,35,38,44,46,47^, as well as the HPV^−^/p16^−^ and the HPV^−^/p16^+^ (fixed effects model; RR of 0.83; 95% CI 0.71–0.96; P = 0.28)^24,32,35,38,42,44,46,47^ showed comparable results to the meta-analyses with all data included (Fig. 2a).

We next performed sensitivity analyses to investigate the effect of additional studies. The HPV^+^/p16^+^ subgroup was associated with better survival compared to the HPV^-^/p16^−^ (fixed effects model; RR of 3.08; 95% CI 2.69–3.51; P = 0.49)^38,43,46,47^, the HPV^−^/p16^+^ (fixed effects model; RR of 2.35; 95% CI 1.88–2.94; P = 0.13)^38,44,46,47^ and the HPV^+^/p16^−^ (random effects model; RR of 2.41; 95% CI 1.44–4.03; P = 0.01)^43–47^. The 5-year OS of the HPV^−^/p16^−^ subgroup was inferior compared to the HPV^−^/p16^+^ (fixed effects model; RR of 0.76; 95% CI 0.62–0.92; P = 0.35)^38,43,46,47^ while the survival of the HPV^+^/p16^−^ was not significantly different (fixed effects model; RR of 0.90; 95% CI 0.78–1.04; P = 0.60)^38,42,44,46,47^. The 5-year OS of the subgroup HPV^+^/p16^−^ and HPV^−^/p16^+^ was not statistically significant (random effects model; RR of 1.09; 95%CI 0.59–1.99; P = 0.04), even when the Ramshankar *et al*. study^42^ was excluded (random effects model; RR of 0.84; 95%CI 0.65–1.08; P = 0.69)^38,44,46,47^. Therefore, the sub meta-analyses confirmed the whole meta-analyses results with the complete international studies data set (Figs 2–3).

### Publication bias

The funnel plot shapes did not reveal obvious evidence of asymmetry.

## Discussion

The incidence of high-risk-HPV^+^ tumors is exceeding 25% in HNSCC and 70% in oropharyngeal HNSCC^48^. This etiologically distinct HNSCC subtype has been associated with improved clinical outcome. Therefore, positive HPV status is a recommended biomarker for patient stratification towards de-escalation treatment regimens. Consequently, a new staging algorithm for OPSCC was recommended recently in the 8th AJCC/UICC guideline. Nevertheless, the strategy for a new staging paradigm in all head and neck regions is still under-investigation and being discussed. Since HPV can be found as an innocent bystander and p16 can be positive independently of HPV, before inclusion into such trials, it should be verified if the tumor is truly HPV-driven in order not to skew data and to avoid undertreatment of the patients’ cancer. In a number of investigations, this discrepancy among HPV markers (p16^+^ IHC and HPV-DNA^+^) was consistently found. And, moreover, these subtypes based on HPV/p16 status have shown different clinical outcomes^21^. In this meta-analysis, we confirm and expand to recent investigations to increase the knowledge about (a) the clinical relevance of HPV^+^ HNSCC and OPSCC; (b) the incidence and clinical course of subtypes of HPV^+^ tumors.

There is a discordant group of p16^−^ cases in the HPV^+^ compartment and HPV^−^ cases in the p16^+^ compartment. The number of studies included for evaluation of the discrepant cases of HPV^+^/p16^−^ and HPV^−^/p16^+^ was increased by eight with addition of 428 new patients in this meta-analysis. The relative incidences of HPV^+^/p16^−^ and HPV^−^/p16^+^ HNSCC were 7.3% and 6.7%, respectively. The survival data showed that HNSCC patients with HPV^+^ status defined as HPV^+^/p16^+^ have a better 5-year OS and DFS than subgroups with HPV^−^/p16^−^, HPV^+^/p16^−^ and HPV^−^/p16^+^. These significant observations have been made in all head and neck regions and in OPSCC in particular and were consistent with previous studies. The sensitivity analysis confirmed the consistency of the 8 new additional studies. Thus, the survival benefit of the HPV^+^/p16^+^ subgroup is obvious. HPV^−^/p16^+^ HNSCC have a better 5-year OS than the HPV^+^/p16^−^ subtype after excluding the study from Ramshankar *et al*.^42^ who found in early staged oral tongue squamous cell carcinoma patients that p16 overexpression was associated with lower survival and increased risk for disease recurrence irrespective of the HPV16 DNA status. Compared to the HPV^−^/p16^−^ subgroup, 5-year OS of HPV^−^/p16^+^ HNSCC is better while HPV^+^/p16^−^ is not. HPV^+^ in p16^−^ HNSCC may be an innocent bystander with no functional involvement. Therefore, a careful investigation is required why HPV is negative to exclude false negative results of HPV tests or to prove truly HPV-independent development. Better survival in p16+ subgroups raises the question for its cause if independent of HPV. Consequently, in clinical trials these subtypes should be investigated separately to clarify if cancers displaying the HPV^−^/p16^+^ phenotype also qualify to be considered for de-escalation protocols.

p16 is a member of the INK4 class of cell-cycle inhibitors (INK4a) and functions as tumor suppressor. It binds to cyclin-dependent-kinases (CDK) 4 and CDK6 and prevents their association with cyclin D1, and consequently, the phosphorylation and inactivation of the retinoblastoma protein (Rb)^49^. In HPV-driven tumors, high-risk HPV E7 protein triggers a cellular defense response mediated by p16 and inactivating the retinoblastoma (Rb) pathway. Therefore, p16 overexpression is an excellent biomarker for high-risk HPV-associated malignancies including cervical cancer^50^ and HNSCC^21^. In a number of premalignant lesions and non-HPV driven tumors^21^, however, a p16 overexpression is also present. The underlying mechanisms of p16 overexpression in these non-HPV driven tumors is currently undetermined. These tumors often harbor mutations such as RAS and BRAF^51^. But a study in p16^+^/HPV^−^ head and neck and anogenital SCCs has shown that overexpression of p16 in these tumors is not an attribute to KRAS mutations^52^. It has been also suggested that deregulation of Rb or Rb loss results in increased p16 expression in tumor cells which is associated with uncontrolled cell proliferation in malignant tumors^53,54^. A recent study identified in HPV^−^ high-grade neuroendocrine carcinomas of the head and neck an overexpression of p16^55^. Most of these tumors had Rb loss and a low or absent cyclin D1 expression. Therefore, we hypothesize that mechanisms other than HPV infection may affect the p16-Rb-cyclin D1 pathway and induce cell cycle activation in HPV^−^ HNSCC. However, future studies are required to clarify the pathogenic mechanisms between these subgroups.

As p53 is a key event in transformation and not directly associated with p16, p53 wild-type has been shown in HPV^+^ tumors and p53 mutation-type staining in HPV^−^ tumors. However, in a cervical adenocarcinoma, diffuse p16 immunoreactivity is not necessarily indicative of a high-risk HPV-associated tumor^56^. p16^INK4A^ enhances the transcriptional and the apoptotic functions of p53 through DNA-dependent interaction^57^. Therefore, we hypothesize that in the HPV^−^/p16^+^ subgroup, which are E6 negative, the p53 is presumably mutated and this subgroup represents the HPV independent cases.

The prevalence of HPV-associated HNSCC potentially depends on the sensitivity and specificity of the detection method. Therefore, the detection technique for HPV may be another cause for discordant results in HPV/p16 testing. Therefore, we analysed the data separately according to the HPV-detection method used. HPV detection by PCR and by ISH only (without PCR) were used as HPV detection methods in 20 and 5 studies, respectively. Sensitivity analysis showed that the results of the 5-year OS were comparable using both HPV detection methods. Evans *et al*.^32^ discussed that HPV testing was performed regardless of DNA quality which may have revealed a high false negative rate in DNA-based HPV detection methods (PCR and ISH). DNA degradation does not have any effect on p16 IHC testing results^32^. To determine the HPV status, Zafereo *et al*. presented an algorithm of p16 immunohistochemistry and HPV ISH and PCR^58^. Ou *et al*. demonstrated an algorithm consisting of two PCR assays and p16 immunohistochemistry^59^. Prigge *et al*. demonstrated in a meta-analysis the high sensitivity but only moderate specificity of p16INK4a and HPV DNA PCR when used as single tests to detect a transforming HPV infection in OPSCC. However, by combining the two tests, specificity was significantly optimized without altering the sensitivity^6^.

During HPV infection, the HPV E6/E7 oncogenes are expressed at low levels. Sensitive techniques such as qPCR detect HPV E6/E7 transcripts despite very limited expression^60–64^. Immunohistochemical evaluation of E6/E7 oncoprotein expression is another method for HPV detection which is independent from RNA or DNA degradation^65^. False negative results in HPV PCR testing may be caused by gene losses when L1 targets were used (15 of 20 studies testing for HPV DNA). These gene losses can cause the PCR to be negative, even though HPV is present (false HPV^−^/p16^+^). For E6/E7 targets this is not the case. However, studies using E6/E7 targets also detected patients belonging to the HPV^−^/p16^+^ subgroup^26,33,34,45,46^. Therefore, the HPV^−^/p16^+^ subgroup should be tested using the uniform high-sensitivity method for E6/E7 expression.

Detection of E6 and E7 mRNA expression is highly associated with p16 expression^60–64^. To identify a truly driven HPV-infection of OPSCC, the RNAscope HPV-test showed comparable results with p16-based algorithms combined with HPV PCR or HPV ISH. The RNAscope HPV-test performed better than p16 alone^66^. The quality of the results using mRNA detection are still controversial^67^. As a first step to analyze causal oncogenic HPV involvement, the detection of viral DNA is more practicable as viral RNA is sensitive to degradation^68^.

In about 90% of HPV-associated OPSCC, the high-risk-HPV-type 16 is found^48,69–71^. Other HPV types than genotype 16 may explain the identified subgroups if HPV tests have a restricted genotype spectrum. Most studies investigated multiple HPV types, however the number of investigated HPV types varied. Some studies investigated only p16^34,37^. After excluding studies which tested only individual HPV types or used HPV detection methods with low sensitivity, we confirmed the distinct 5- year OS of the HPV^−^/p16^+^ subgroup to be inferior compared with the HPV^+^/p16^+^ subgroup and superior compared with the HPV^−^/p16^−^ subgroup. Thus, the HPV^−^/p16^+^ subgroup should be tested for all possibly involved HPV genotypes. In addition, the HPV^−^/p16^+^ subgroup may be caused by HPV independent mechanisms.

HPV^+^ OPSCCs have similar survival benefits in Brazil (GENCAPO study), the US (CHANCE study), and Europe (ARCAGE study)^72^. The present sensitivity analysis showed comparable results for the 5-year OS in Europe, the US, and Asia. Therefore, a geographic differentiation of the study origin is not necessary. As anti-smoking campaigns and their success differ in these countries overall there is a difference in the portion of the HPV positive HNSCC. However, HPV^+^ patients with tobacco consumption have to be distinguished from HPV^+^ non-smokers^73–75^ because the true etiology may be drug-associated and HPV an innocent bystander infection.

The prognostic utility of HPV among non-oropharyngeal-derived HNSCC is limited. The effect of HPV16/p16 was significantly different in non-OPSCC compared with OPSCC^72^. Chung *et al*.^40^ found that p16^+^ non-OPSCC have better outcomes compared to the corresponding patients with p16^−^ non-OPSCC. Salazar *et al*.^37^ found no survival benefit for non-OPSCC in p16^+^ patients. However, when both p16 and HPV DNA were considered, concordantly positive non-OPSCC had significantly better survival. There were not sufficient data to perform a meta-analysis for non-OPSCC in the present study, therefore, a definitive conclusion cannot be drawn at this time.

After the recent introduction of a specific TNM system for HPV^+^ cancers and trials evaluating de-escalation protocols, HPV detection that includes detection of activity by measuring mRNA and protein of HPV oncogenes is an important step to correctly interpret data: HPV oncogene expression is prognosis relevant while HPV DNA as a bystander is not. One example for such a misinterpretation that is under current circumstances possible is p16^+^HNSCC with HPV bystander infection that results in a HPV^+^/p16^+^ phenotype which would possibly be undertreated in a de-escalation protocol. Therefore, as an ideal detection method for HPV-associated/driven cancers we propose the following algorithm for detection: perform IHC to determine the p16-status and if the staining is positive, determine positivity for HPV-oncogene mRNA or protein.

In conclusion, the information obtained from our meta analysis has revealed a potential new biologic subtype of HNSCC. From a research and clinical perspective, recruiting HPV-driven patients would be critical for the success of clinical trials towards redirecting the treatment of those patients. Further undertreatment in cases of bystander HPV and HPV-independent p16^+^ needs to be avoided. It remains important to elucidate the risk for progression and therapy failure especially for the HPV^−^/p16^+^ subgroup and potentially HPV false negative patients to prevent erroneous classification of patients for downscaling treatment by de-escalation-therapy. Additionally, re-examining patient’s specimens in a cluster by testing HPV-oncogene mRNA or protein can identify additional epidemiologic links between HPV-driven and non-HPV-driven tumors.
